# Comparison Between Intravenous Urography and Computed Tomography Urography in Diagnosing Ureteropelvic Junction Obstruction

**DOI:** 10.5812/numonthly.3402

**Published:** 2012-06-20

**Authors:** Cevdet Kaya, Selahattin Çalışkan

**Affiliations:** 1 Haydarpaşa Numune Training and Research Hospital, Istanbul, Turkey

**Keywords:** Ureter, Urography, kidney

Dear Editor,

We read with interest the article by Xie et al. (1) in a previous issue of Nephro-Urology Monthly (October 2011), regarding the comparison between intravenous urography and computed tomography urography in diagnosing ureteropelvic junction obstruction.

As previously described, pathological lesions at the ureteropelvic junction are classified into intrinsic and extrinsic abnormalities. In the case of intrinsic pathology, increased collagen and ground substances between the muscle bundles have been demonstrated in many studies (2). Additionally we have recently found that intrinsic UPJ obstruction samples have a dissolved smooth muscular coat and an overexpression of extracellular matrix proteins, together with a depleted nerve supply (3). Hypoplastic adynamic ureteral segment was also recently shown to be the cause of the lack of adjustment to increased workloads (high urine volume). Ureteral bands and kinks were defined, especially in older children with symptomatic Ureteropelvic junction obstruction obstruction (4).

Although the authors compared intravenous pyelography with computed tomography urography in this paper, we should not forget the importance of diuretic renography as a noninvasive test, to determine the severity and functional significance of ureteropelvic urine transport problems in children. This test is the most widely used one and it is very helpful in deciding the final result, whether the operation is essential or not, before the operation. Some centers have pointed out that their current imaging modality of choice is CT urography, which is particularly useful in showing the anatomy of aberrant vessels, secondary kinks and adhesions (4). Associated renal anomalies (horse-shoe kidney, duplex kidney etc.) can be clearly demonstrated as well with a comparable radiation exposure to standard intravenous pyelogram.
